# Coefficient Estimates for New Subclasses of Meromorphic Bi-Univalent Functions

**DOI:** 10.1155/2014/376076

**Published:** 2014-10-28

**Authors:** Serap Bulut

**Affiliations:** Civil Aviation College, Kocaeli University, Arslanbey Campus, İzmit, 41285 Kocaeli, Turkey

## Abstract

We introduce and investigate two new subclasses *ℳ*_*σ*_(*α*, *λ*) and *ℳ*_*σ*_(*β*, *λ*) of meromorphic bi-univalent functions defined on Δ = {*z* : *z* ∈ *ℂ*, 1 < |*z* | <*∞*}. For functions belonging to these classes, estimates on the initial coefficients are obtained.

## 1. Introduction

Let *𝒜* denote the class of all functions of the form

(1) $$\begin{array}{c} f\left(z\right)=z+\overset{\infty }{\underset{k=2}{\sum }}a_{k}z^{k}, \end{array}$$

which are analytic in the open unit disk

(2) $$\begin{array}{c} U=\left\{z:z\in C,\left|z\right|<1\right\}. \end{array}$$

We also denote by *𝒮* the class of all functions in the normalized analytic function class *𝒜* which are univalent in *𝕌*.

Since univalent functions are one-to-one, they are invertible and the inverse functions need not be defined on the entire unit disk *𝕌*. In fact, the Koebe one-quarter theorem [1] ensures that the image of *𝕌* under every univalent function *f* ∈ *𝒮* contains a disk of radius 1/4. Thus, every function *f* ∈ *𝒜* has an inverse *f* ^−1^, which is defined by

(3) $$\begin{array}{c} f^{-1}\left(f\left(z\right)\right)=z\;\left(z\in U\right),\\ f\left(f^{-1}\left(w\right)\right)=w\;\left(\left|w\right|<r_{0}\left(f\right);r_{0}\left(f\right)\geq \frac{1}{4}\right). \end{array}$$

In fact, the inverse function *f* ^−1^ is given by

(4) f−1(w)=w−a2w2+(2a22−a3)w3 −(5a23−5a2a3+a4)w4+⋯.

A function *f* ∈ *𝒜* is said to be bi-univalent in *𝕌* if both *f* and *f* ^−1^ are univalent in *𝕌*. Let Σ denote the class of bi-univalent functions in *𝕌* given by (1). For a brief history and interesting examples of functions in the class Σ, see [2] (see also [3, 4]). In fact, the aforecited work of Srivastava et al. [2] essentially revived the investigation of various subclasses of the bi-univalent function class Σ in recent years; it was followed by such works as those by Murugusundaramoorthy et al. [5], Frasin and Aouf [6], Çağlar et al. [7], and others (see, e.g., [8–15]).

In this paper, the concept of bi-univalency is extended to the class of meromorphic functions defined on

(5) $$\begin{array}{c} \Delta =\left\{z:z\in C,1<\left|z\right|<\infty \right\}. \end{array}$$

For this purpose, let *σ* denote the class of all meromorphic univalent functions *g* of the form

(6) $$\begin{array}{c} g\left(z\right)=z+\overset{\infty }{\underset{n=0}{\sum }}\frac{b_{n}}{z^{n}}, \end{array}$$

defined on the domain Δ. Since *g* ∈ *σ* is univalent, it has an inverse *g* ^−1^ that satisfies

(7) $$\begin{array}{c} g^{-1}\left(g\left(z\right)\right)=z\;\left(z\in \Delta \right),\\ g\left(g^{-1}\left(w\right)\right)=w\;\left(M<\left|w\right|<\infty ;M>0\right). \end{array}$$

Furthermore, the inverse function *g* ^−1^ has a series expansion of the form

(8) $$\begin{array}{c} g^{-1}\left(w\right)=w+\overset{\infty }{\underset{n=0}{\sum }}\frac{B_{n}}{w^{n}}, \end{array}$$

where *M* < |*w*| < *∞*. Analogous to the bi-univalent analytic functions, a function *g* ∈ *σ* is said to be meromorphically bi-univalent if both *g* and *g* ^−1^ are meromorphically univalent in Δ. We denote by *σ*_*ℳ*_ the class of all meromorphic bi-univalent functions in Δ given by (6). A simple calculation shows that

(9) $$\begin{array}{c} g^{-1}\left(w\right)=w-b_{0}-\frac{b_{1}}{w}-\frac{b_{2}+b_{0}b_{1}}{w^{2}}-\cdots . \end{array}$$

The coefficient problem was investigated for various interesting subclasses of the meromorphic univalent functions (see, e.g., [16–18]).

In the present investigation, certain subclasses of meromorphic bi-univalent functions are introduced and estimates for the coefficients *b*_0_ and *b*_1_ of functions in the newly introduced subclasses are obtained.

**Definition 1 .**

A function *g* given by (6) is said to be in the class *ℳ*_*σ*_(*α*, *λ*) if the following conditions are satisfied:

(10) g∈σM,  |arg(zg′(z)(1−λ)g(z)+λzg′(z))|<απ2mmmmmmmmmml(0<α≤1,0≤λ<1,z∈Δ),    |arg(wh′(w)(1−λ)h(w)+λwh′(w))|<απ2    mmmmm(0<α≤1,0≤λ<1,w∈Δ),

where the function *h* = *g* ^−1^ is given by (9).

**Definition 2 .**

A function *g* given by (6) is said to be in the class *ℳ*_*σ*_(*β*, *λ*) if the following conditions are satisfied:

(11) g∈σM,  R{zg′(z)(1−λ)g(z)+λzg′(z)}>βmmmmmmmml(0≤β<1,0≤λ<1,z∈Δ),    R{wh′(w)(1−λ)h(w)+λwh′(w)}>β    mml(0≤β<1,0≤λ<1,w∈Δ),

where the function *h* = *g* ^−1^ is given by (9).

**Remark 3 .**

We note that, for *λ* = 0, the classes *ℳ*_*σ*_(*α*, *λ*) and *ℳ*_*σ*_(*β*, *λ*) reduce to the classes

(12) $$\begin{array}{c} M_{\sigma }\left(\alpha ,0\right)={\tilde{\Sigma }}_{B}^{*}\left(\alpha \right),\\ M_{\sigma }\left(\beta ,0\right)=\Sigma_{B}^{*}\left(\beta \right), \end{array}$$

respectively, introduced and studied by Halim et al. [17].

The object of the present paper is to extend the concept of bi-univalent to the class of meromorphic functions defined on Δ and find estimates on the coefficients |*b*_0_| and |*b*_1_| for functions in the above-defined classes *ℳ*_*σ*_(*α*, *λ*) and *ℳ*_*σ*_(*β*, *λ*) of the function class *σ*_*ℳ*_.

Firstly, in order to derive our main results, we need the following lemma.

**Lemma 4 (see [19]).**

If *p* ∈ *𝒫*, then |*c*_*k*_| ≤ 2 for each *k*, where *𝒫* is the family of all functions *p* analytic in *𝕌* for which

(13) $$\begin{array}{c} R\left(p\left(z\right)\right)>0,\;p\left(z\right)=1+c_{1}z+c_{2}z^{2}+\cdots , \end{array}$$

for *z* ∈ *𝕌*.

## 2. Coefficient Estimates

We begin this section by finding the estimates on the coefficients |*b*_0_| and |*b*_1_| for functions in the class *ℳ*_*σ*_(*α*, *λ*).

**Theorem 5 .**

Let the function *g*(*z*) given by the series expansion (6) be in the function class

(14) $$\begin{array}{c} M_{\sigma }\left(\alpha ,\lambda \right)\;\left(0<\alpha \leq 1,0\leq \lambda <1\right). \end{array}$$

Then

(15) $$\begin{array}{c} \left|b_{0}\right|\leq \frac{\sqrt{2}\alpha }{1-\lambda }, \end{array}$$

(16) $$\begin{array}{c} \left|b_{1}\right|\leq \{\begin{array}{cc} \frac{\sqrt{2}\alpha^{2}}{1-\lambda }, & 0<\alpha \leq \frac{1}{\sqrt{2}},\\ \frac{\alpha }{1-\lambda }, & \frac{1}{\sqrt{2}}\leq \alpha \leq 1. \end{array} \end{array}$$

**Proof**

It follows from (10) that

(17) $$\begin{array}{c} \frac{zg^{\prime }\left(z\right)}{\left(1-\lambda \right)g\left(z\right)+\lambda zg^{\prime }\left(z\right)}={\left[p\left(z\right)\right]}^{\alpha }\;\left(z\in \Delta \right),\\ \frac{wh^{\prime }\left(w\right)}{\left(1-\lambda \right)h\left(w\right)+\lambda wh^{\prime }\left(w\right)}={\left[q\left(w\right)\right]}^{\alpha }\;\left(w\in \Delta \right), \end{array}$$

respectively, where *p*(*z*) and *q*(*w*) are functions with positive real part in Δ and have the forms

(18) $$\begin{array}{c} p\left(z\right)=1+\frac{p_{1}}{z}+\frac{p_{2}}{z^{2}}+\cdots , \end{array}$$

(19) $$\begin{array}{c} q\left(w\right)=1+\frac{q_{1}}{w}+\frac{q_{2}}{w^{2}}+\cdots , \end{array}$$

respectively. Now, upon equating the coefficients in (17), we get

(20) $$\begin{array}{c} -\left(1-\lambda \right)b_{0}=\alpha p_{1}, \end{array}$$

(21) $$\begin{array}{c} \left(1-\lambda \right)\left[\left(1-\lambda \right)b_{0}^{2}-2b_{1}\right]=\alpha p_{2}+\frac{\alpha \left(\alpha -1\right)}{2}p_{1}^{2}, \end{array}$$

(22) $$\begin{array}{c} \left(1-\lambda \right)b_{0}=\alpha q_{1}, \end{array}$$

(23) $$\begin{array}{c} \left(1-\lambda \right)\left[\left(1-\lambda \right)b_{0}^{2}+2b_{1}\right]=\alpha q_{2}+\frac{\alpha \left(\alpha -1\right)}{2}q_{1}^{2}. \end{array}$$

From (20) and (22), we find that

(24) $$\begin{array}{c} p_{1}=-q_{1}, \end{array}$$

(25) $$\begin{array}{c} 2{\left(1-\lambda \right)}^{2}b_{0}^{2}=\alpha^{2}\left(p_{1}^{2}+q_{1}^{2}\right). \end{array}$$

Also, from (21) and (23) we obtain

(26) $$\begin{array}{c} 2{\left(1-\lambda \right)}^{2}b_{0}^{2}=\alpha \left(p_{2}+q_{2}\right)+\frac{\alpha \left(\alpha -1\right)}{2}\left(p_{1}^{2}+q_{1}^{2}\right). \end{array}$$

Since *ℜ*(*p*(*z*)) > 0 and *ℜ*(*q*(*z*)) > 0 in Δ, the functions *p*(1/*z*), *q*(1/*z*) ∈ *𝒫* and hence the coefficients *p*_*k*_ and *q*_*k*_ for each *k* satisfy the inequality in Lemma 4. Applications of triangle inequality followed by Lemma 4 in (25) and (26) give us the required estimates on |*b*_0_| as asserted in (15).

Next, in order to find the bound on the coefficient |*b*_1_|, we subtract (23) from (21). We thus get

(27) $$\begin{array}{c} -4\left(1-\lambda \right)b_{1}=\alpha \left(p_{2}-q_{2}\right). \end{array}$$

Hence

(28) $$\begin{array}{c} \left|b_{1}\right|\leq \frac{\alpha }{1-\lambda }. \end{array}$$

On the other hand, using (21) and (23) yields

(29) 2(1−λ)2[(1−λ)2b04+4b12]  =α2(α−1)24(p14+q14)+α2(p22+q22)   +α2(α−1)(p12p2+q12q2).

By using (25) we have from the above equality

(30) b12=α2(α−1)232(1−λ)2(p14+q14)+α28(1−λ)2(p22+q22) +α2(α−1)8(1−λ)2(p12p2+q12q2) −α416(1−λ)2(p14+q14)−α48(1−λ)2p12q12.

From Lemma 4, we obtain

(31) $$\begin{array}{c} \left|b_{1}\right|\leq \frac{\sqrt{5}\alpha^{2}}{1-\lambda }. \end{array}$$

Also, by using (26) we have, from equality (29),

(32) $$\begin{array}{c} \left|b_{1}\right|\leq \frac{\sqrt{2}\alpha^{2}}{1-\lambda }. \end{array}$$

Comparing (28), (31), and (32) we get the desired estimate on the coefficient |*b*_1_| as asserted in (16).

For *λ* = 0, we have the following corollary of Theorem 5.

**Corollary 6 .**

Let the function *g*(*z*) given by the series expansion (6) be in the function class

(33) $$\begin{array}{c} {\tilde{\Sigma }}_{B}^{*}\left(\alpha \right)\;\left(0<\alpha \leq 1\right). \end{array}$$

Then

(34) $$\begin{array}{c} \left|b_{0}\right|\leq \sqrt{2}\alpha ,\\ \left|b_{1}\right|\leq \{\begin{array}{cc} \sqrt{2}\alpha^{2}, & 0<\alpha \leq \frac{1}{\sqrt{2}},\\ \alpha , & \frac{1}{\sqrt{2}}\leq \alpha \leq 1. \end{array} \end{array}$$

**Remark 7 .**

Corollary 6 is an improvement of the following estimates which were given by Halim et al. [17].

**Corollary 8 (see [17]).**

Let the function *g*(*z*) given by the series expansion (6) be in the function class

(35) $$\begin{array}{c} {\tilde{\Sigma }}_{B}^{*}\left(\alpha \right)\;\left(0<\alpha \leq 1\right). \end{array}$$

Then

(36) $$\begin{array}{c} \left|b_{0}\right|\leq 2\alpha ,\\ \left|b_{1}\right|\leq \sqrt{5}\alpha^{2}. \end{array}$$

**Remark 9 .**

Corollary 6 is also an improvement of the estimates which were given by Panigrahi [20, Corollary 2.3].

Next we estimate the coefficients |*b*_0_| and |*b*_1_| for functions in the class *ℳ*_*σ*_(*β*, *λ*).

**Theorem 10 .**

Let the function *g*(*z*) given by the series expansion (6) be in the function class

(37) $$\begin{array}{c} M_{\sigma }\left(\beta ,\lambda \right)\;\left(0\leq \beta <1,0\leq \lambda <1\right). \end{array}$$

Then

(38) $$\begin{array}{c} \left|b_{0}\right|\leq \frac{\sqrt{2\left(1-\beta \right)}}{1-\lambda }, \end{array}$$

(39) $$\begin{array}{c} \left|b_{1}\right|\leq \frac{1-\beta }{1-\lambda }. \end{array}$$

**Proof**

It follows from (11) that

(40) $$\begin{array}{c} \frac{zg^{\prime }\left(z\right)}{\left(1-\lambda \right)g\left(z\right)+\lambda zg^{\prime }\left(z\right)}=\beta +\left(1-\beta \right)p\left(z\right)\;\left(z\in \Delta \right),\\ \frac{wh^{\prime }\left(w\right)}{\left(1-\lambda \right)h\left(w\right)+\lambda wh^{\prime }\left(w\right)}=\beta +\left(1-\beta \right)q\left(w\right)\;\left(w\in \Delta \right), \end{array}$$

respectively, where *p*(*z*) and *q*(*w*) are functions with positive real part in Δ and have the forms (18) and (19), respectively. Now, upon equating the coefficients in (40), we get

(41) $$\begin{array}{c} -\left(1-\lambda \right)b_{0}=\left(1-\beta \right)p_{1}, \end{array}$$

(42) $$\begin{array}{c} \left(1-\lambda \right)\left[\left(1-\lambda \right)b_{0}^{2}-2b_{1}\right]=\left(1-\beta \right)p_{2}, \end{array}$$

(43) $$\begin{array}{c} \left(1-\lambda \right)b_{0}=\left(1-\beta \right)q_{1}, \end{array}$$

(44) $$\begin{array}{c} \left(1-\lambda \right)\left[\left(1-\lambda \right)b_{0}^{2}+2b_{1}\right]=\left(1-\beta \right)q_{2}. \end{array}$$

From (41) and (43), we obtain

(45) $$\begin{array}{c} p_{1}=-q_{1}, \end{array}$$

(46) $$\begin{array}{c} 2{\left(1-\lambda \right)}^{2}b_{0}^{2}={\left(1-\beta \right)}^{2}\left(p_{1}^{2}+q_{1}^{2}\right). \end{array}$$

Also, from (42) and (44), we obtain

(47) $$\begin{array}{c} 2{\left(1-\lambda \right)}^{2}b_{0}^{2}=\left(1-\beta \right)\left(p_{2}+q_{2}\right). \end{array}$$

Since *ℜ*(*p*(*z*)) > 0 and *ℜ*(*q*(*z*)) > 0 in Δ, the functions *p*(1/*z*), *q*(1/*z*) ∈ *𝒫* and hence the coefficients *p*_*k*_ and *q*_*k*_ for each *k* satisfy the inequality in Lemma 4. Therefore, we find from (46) and (47) that

(48) $$\begin{array}{c} \left|b_{0}\right|\leq \frac{2\left(1-\beta \right)}{1-\lambda },\\ \left|b_{0}\right|\leq \frac{\sqrt{2\left(1-\beta \right)}}{1-\lambda }, \end{array}$$

respectively. So we get the desired estimate on the coefficient |*b*_0_| as asserted in (38).

Next, in order to find the bound on the coefficient |*b*_1_|, we subtract (44) from (42). We thus get

(49) $$\begin{array}{c} -4\left(1-\lambda \right)b_{1}=\left(1-\beta \right)\left(p_{2}-q_{2}\right). \end{array}$$

Hence

(50) $$\begin{array}{c} \left|b_{1}\right|\leq \frac{1-\beta }{1-\lambda }. \end{array}$$

On the other hand, using (42) and (44) yields

(51) $$\begin{array}{c} {\left(1-\lambda \right)}^{2}\left[{\left(1-\lambda \right)}^{2}b_{0}^{4}-4b_{1}^{2}\right]={\left(1-\beta \right)}^{2}p_{2}q_{2} \end{array}$$

or equivalently

(52) $$\begin{array}{c} 4b_{1}^{2}={\left(1-\lambda \right)}^{2}b_{0}^{4}-\frac{{\left(1-\beta \right)}^{2}}{{\left(1-\lambda \right)}^{2}}p_{2}q_{2}. \end{array}$$

Upon substituting the value of *b*_0_^2^ from (46) and (47) into (52), respectively, it follows that

(53) $$\begin{array}{c} \left|b_{1}\right|\leq \frac{1-\beta }{1-\lambda }\sqrt{4\beta^{2}-8\beta +5},\\ \left|b_{1}\right|\leq \frac{\sqrt{2}\left(1-\beta \right)}{1-\lambda }. \end{array}$$

Comparing (50) and (53), we get the desired estimate on the coefficient |*b*_1_| as asserted in (39).

For *λ* = 0, we have the following corollary of Theorem 10.

**Corollary 11 .**

Let the function *g*(*z*) given by the series expansion (6) be in the function class

(54) $$\begin{array}{c} \Sigma_{B}^{*}\left(\beta \right)\;\left(0\leq \beta <1\right). \end{array}$$

Then

(55) $$\begin{array}{c} \left|b_{0}\right|\leq \sqrt{2\left(1-\beta \right)},\\ \left|b_{1}\right|\leq 1-\beta . \end{array}$$

**Remark 12 .**

Corollary 11 is an improvement of the following estimates which were given by Halim et al. [17].

**Corollary 13 (see [17]).**

Let the function *g*(*z*) given by the series expansion (6) be in the function class

(56) $$\begin{array}{c} \Sigma_{B}^{*}\left(\beta \right)\;\left(0\leq \beta <1\right). \end{array}$$

Then

(57) $$\begin{array}{c} \left|b_{0}\right|\leq 2\left(1-\beta \right),\\ \left|b_{1}\right|\leq \left(1-\beta \right)\sqrt{4\beta^{2}-8\beta +5}. \end{array}$$

**Remark 14 .**

Corollary 11 is also an improvement of the estimates which were given by Panigrahi [20, Corollary 3.3].

## References

[1] Duren P. L. (1983). Univalent functions. *Grundlehren der Mathematischen Wissenschaften*.

[2] Srivastava H. M., Mishra A. K., Gochhayat P. (2010). Certain subclasses of analytic and bi-univalent functions. *Applied Mathematics Letters*.

[3] Brannan D. A., Taha T. S., Mazhar S. M., Hamoui A., Faour N. S. (1988). On some classes of bi-univalent functions. *Mathematical Analysis and Its Applications : Proceedings of the International Conference on Mathematical Analysis and Its Applications, Safat, Kuwait 18–21 February 1985*.

[4] Brannan D. A., Taha T. S. (1986). On some classes of bi-univalent functions. *Studia Universitatis Babes-Bolyai. Mathematica*.

[5] Murugusundaramoorthy G., Magesh N., Prameela V. (2013). Coefficient bounds for certain subclasses of bi-univalent function. *Abstract and Applied Analysis*.

[6] Frasin B. A., Aouf M. K. (2011). New subclasses of bi-univalent functions. *Applied Mathematics Letters*.

[7] Çağlar M., Orhan H., Yağmur N. (2013). Coefficient bounds for new subclasses of bi-univalent functions. *Filomat*.

[8] Bulut S. (2013). Coefficient estimates for a class of analytic and bi-univalent functions. *Novi Sad Journal of Mathematics*.

[9] Bulut S. (2013). Coefficient estimates for new subclasses of analytic and bi-univalent functions defined by Al-Oboudi differential operator. *Journal of Function Spaces and Applications*.

[10] Bulut S. (2013). Coefficient estimates for initial Taylor-Maclaurin coefficients for a subclass of analytic and bi-univalent functions defined by Al-Oboudi differential operator. *The Scientific World Journal*.

[11] Bulut S. Coefficient estimates for a new subclass of analytic and bi-univalent functions.

[12] Hayami T., Owa S. (2012). Coefficient bounds for bi-univalent functions. *Panamerican Mathematical Journal*.

[13] Srivastava H. M., Bulut S., Çağlar M., Yağmur N. (2013). Coefficient estimates for a general subclass of analytic and bi-univalent functions. *Filomat*.

[14] Xu Q., Gui Y., Srivastava H. M. (2012). Coefficient estimates for a certain subclass of analytic and bi-univalent functions. *Applied Mathematics Letters*.

[15] Xu Q.-H., Xiao H.-G., Srivastava H. M. (2012). A certain general subclass of analytic and bi-univalent functions and associated coefficient estimate problems. *Applied Mathematics and Computation*.

[16] Hamidi S. G., Halim S. A., Jahangiri J. M. (2013). Coefficent estimates for bi-univalent strongly starlike and Bazilevic functions. *International Journal of Mathematics Research*.

[17] Halim S. A., Hamidi S. G., Ravichandran V. Coefficient estimates for meromorphic bi-univalent functions.

[18] Janani T., Murugusundaramoorthy G. (2014). Coefficient estimates of meromorphic bi- starlike functions of complex order. *International Journal of Analysis and Applications*.

[19] Pommerenke C. (1975). *Univalent Functions*.

[20] Panigrahi T. (2013). Coefficient bounds for certain subclasses of meromorphic and bi-univalent functions. *Bulletin of the Korean Mathematical Society*.

